# A “Harmless Remedy for Headache, Sleeplessness, Etc.,” Found to Contain Harmful Drug*Office of Information, U. S. Department of Agriculture, Washington, D. C.

**Published:** 1913-10

**Authors:** 


### A "Harmless Remedy for Headache, Sleeplessness, etc.," found to Contain Harmful Drug.*

A "harmless and speedy remedy for headache, neuralgia, sleeplessness, depression, induced by excessive indulgence in liquor, sour stomach, indigestion, nausea, painful menstruation and other nervous disorders" was alleged to be misbranded because it contained a harmful drug, according to a notice of judgment just issued by the Department of Agriculture.

James Vanatta, alias Century Chemical Company, of Indianapolis, Indiana, was charged with the shipment of a quantity of "Celery-Visce" which was described on the label in the above terms. It was also stated to be "pleasant as cream soda."

Having described it as a harmless remedy, the label continues as follows:

Directions: Dash with force a third of a glass of water on a heaping teaspoonful. Drink while foaming. Repeat in 20 minutes if necessary. Acetphenetidin less than four per cent. Compound Vanilla flavoring. Guaranteed under the Pure Food and Drugs Act, June 30, 1906.

According to the view of the Department of Agriculture the statement that the product contains acetphenetidin, is inconsistent with the former statement that the product is harmless.

The printing on the paper wrapper and package containing the product was regarded as false and misleading. The defendant entered a plea of guilty and the court imposed a fine of twenty-five dollars and costs.

*Office of Information, U. S. Department of Agriculture, Washington, D. C.

